# Improving deep convolutional neural networks with mixed maxout units

**DOI:** 10.1371/journal.pone.0180049

**Published:** 2017-07-20

**Authors:** Hui-zhen Zhao, Fu-xian Liu, Long-yue Li

**Affiliations:** Air and Missile Defense College, Air Force Engineering University, Xian, Shaanxi, P.R. China; Tongji University, CHINA

## Abstract

Motivated by insights from the maxout-units-based deep Convolutional Neural Network (CNN) that “non-maximal features are unable to deliver” and “feature mapping subspace pooling is insufficient,” we present a novel mixed variant of the recently introduced maxout unit called a mixout unit. Specifically, we do so by calculating the exponential probabilities of feature mappings gained by applying different convolutional transformations over the same input and then calculating the expected values according to their exponential probabilities. Moreover, we introduce the Bernoulli distribution to balance the maximum values with the expected values of the feature mappings subspace. Finally, we design a simple model to verify the pooling ability of mixout units and a Mixout-units-based Network-in-Network (NiN) model to analyze the feature learning ability of the mixout models. We argue that our proposed units improve the pooling ability and that mixout models can achieve better feature learning and classification performance.

## Introduction

In recent years, the regularization of deep learning models through stochastic model averaging has become an effective tool to ameliorate the overfitting phenomenon in supervised classification tasks[1]. Proposed by Hinton in 2012, dropout became the first model regularization method that uses a stochastic model-averaging technique to improve the performance of deep learning models. The basic idea behind the dropout strategy is to sample half the neurons to act on the output through weighting the full connection by a Bernoulli distribution. The effect of the stochastic neurons makes the classification less reliant on arbitrary units, thus reducing overfitting [2,3]. Krizhevsky applied dropout to several different scale benchmark datasets and verified its good performance [4]. Because the “model-averaging ability” of dropout can greatly improve the performance of a convolutional neural network (CNN), various scholars have proposed a number of improved stochastic model-averaging methods to gain further improvements. Wang sped up the dropout training procedure through a Gaussian approximation method [5]. Ba set the probability of dropout in every hidden layer with a 2-layer belief network, which shared the same parameters with a CNN, to improve the learning effect of the network [6]. Tompson applied dropout to the entire feature space, forming the space-dropout method [7]. Based on dropout, Wan proposed the DropConnect method, which randomly dropped connections between units rather than their activation [8]. Through training unit models that share millions of parameters and average the impact of units on the entire model output, dropout was shown to be effective against overfitting and to improve model feature learning ability. The dropout regime can be viewed as making a significant update to a different model on a different subset in back propagation; therefore, a model combined with dropout appears to perform better when it takes relatively large steps in parameter space. Thus, the ideal regime for dropout is when the overall training procedure resembles training an ensemble with bagging under parameter-sharing constraints [9].

To leverage the approximate model-averaging ability of dropout, Goodfellow designed a novel model called the maxout network [9], which consists of maxout units that can be considered as a new activation function. Instead of using traditional activation functions, a maxout unit takes the maximum feature across several affine feature maps to create a nonlinear transformation. A maxout network with more than two hidden maxout units can approximate any continuous function arbitrarily well on a compact domain. As a universal approximator, the maxout unit describes biological neural characteristics better than do traditional nonlinear activation functions, leading to a better use of the model-averaging technology of dropout. The success of the maxout network can be attributed partly to the fact that maxout units aid the optimization procedure with their continuous piecewise linear ability. Otherwise, their success also depends on the fact that each maxout unit performs a max pooling operation [10] over a subspace of several linear feature mappings. As a result of this subspace pooling operation, a maxout unit selects the maximum feature to represent the inputs [11]. However, while the pooling operation computes the maximum of one feature mapping subspace, non-maximal features are ignored and have no effect on the output. Aiming to improve consideration of the non-maximal features, some other pooling operations have been proposed to replace the maximum operation used in the maxout units. Springenberg proposed a probabilistic maxout (probout) unit to improve the stochastic generalization of maxout units [1]. The probout units preserve the desirable properties of maxout units and improve the subspace pooling operation while increasing the complexity of the model.

Aiming at the problems that “non-maximal features are unable to deliver” and “feature mapping subspace pooling is insufficient,” we present a novel mixed variant of the maxout unit called a mixout unit. By randomly sampling from both the maximum values and expected values of the feature mapping subspaces, our proposed mixout units improve the pooling property. We designed a sample model to verify the pooling ability and a mixout units-based network-in-network (NiN) to analyze the feature learning ability of the mixout units. Experiments on three benchmark datasets show the state-of-the-art performance of the mixout units.

The remainder of this paper is organized as follows. We introduce maxout units and the dropout strategy in Section 2. Our proposed mixout units are presented and analyzed thoroughly in Section 3. We describe experiments and discuss results in Section 4 and provide conclusions in Section 5.

## Related research

In this section, we first introduce the maxout unit and summarize its characteristics. And then, we present the dropout strategy and the “mean network”.

### Maxout units

Given an input vector ***x*** ∈ R^*d*^ to the *l*th hidden layer of a maxout network, the output ***h***(*x*) after this maxout hidden layer takes the maximum across the feature subspace, consisting of *k* affine feature mappings. It is defined as follows:

$$h\left(x\right)=\max\left\{z_{1},\cdots ,z_{k}\right\},$$ (1)

where *z*_*j*_ is a feature map defined as

$$z_{j}=x^{T}W_{j}+b_{j}.$$ (2)

**Fig 1:**
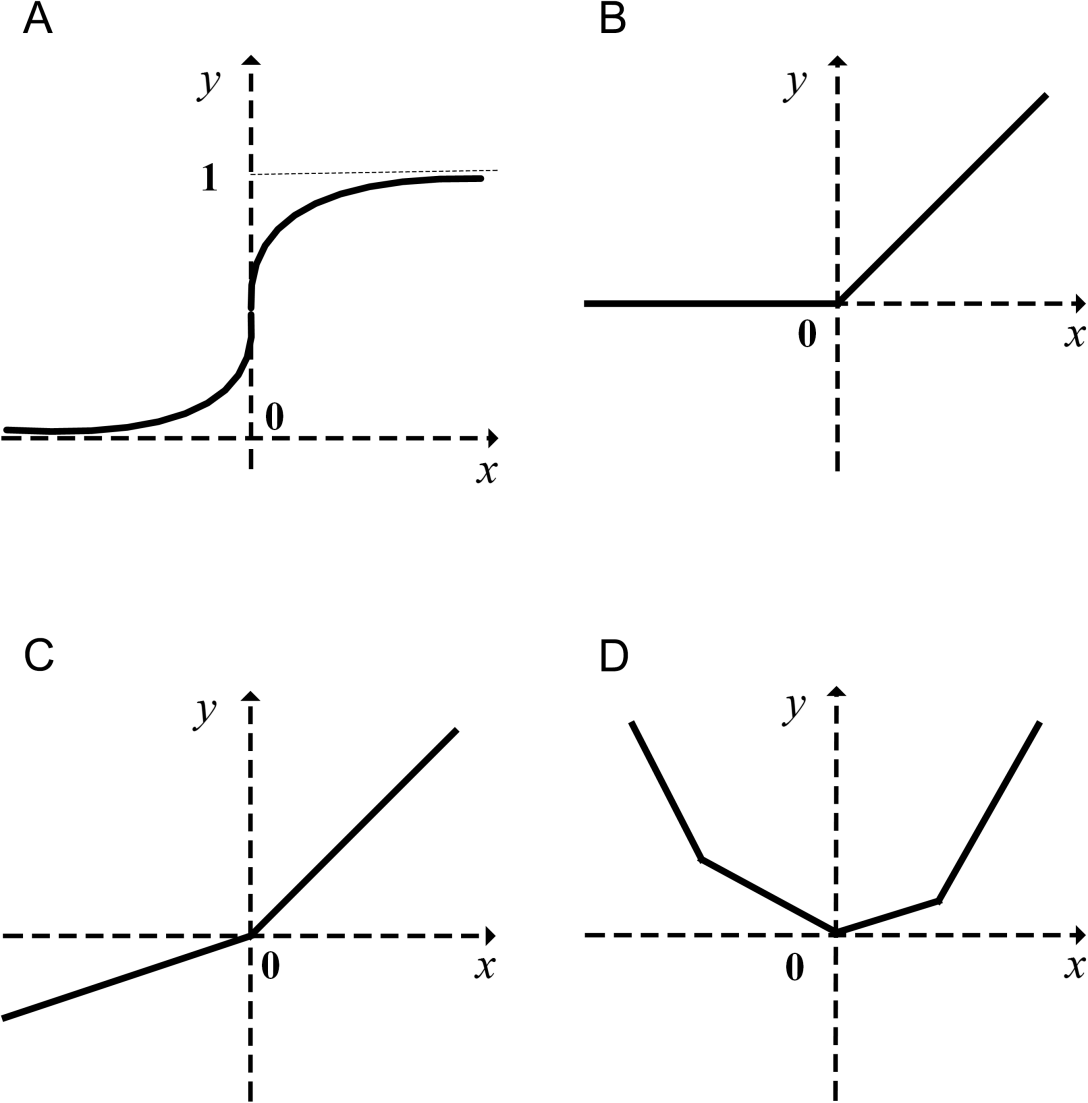
Graphical depiction of some activation functions.

In Eq (2), ***x*** ∈ R^*d*^ is the input vector, and the weight metric ***W*** ∈ R^*d*×*m*×*k*^ and the bias metric ***b*** ∈ R^*m*×*k*^ are all learned though back- propagation. When the input dimension is 1 and the number of affine feature maps in a feature subspace is 4, a graphical depiction of the piecewise maxout unit is shown in Fig 1D. We also show some activation functions: sigmoid in Fig 1A, ReLU in Fig 1B and LReLU in Fig 1C.

The maxout unit can approximate an arbitrary convex function with a continuous piecewise linear ability and pool over the feature subspace, thus improving performance.

### Dropout strategy

The model-averaging techniques can balance the influence of each unit in a model on the output and reduce the influence of one another’s complex cooperation. Such general features of the model can be learned. If the synergy of two units has an impact on the output of the model in the training process, the model will adjust its parameters to study the symbiotic relationship between the two units and how it is prone to overfitting. Thus, the models cannot learn the real information of the input data if the model units are not balanced.

Dropout is a strategy that can regulate the CNN models and greatly improve the performance by using stochastic model-averaging techniques. Given a deep learning model with *L*−1 hidden layers and one softmax layer, the output of the *l*th layer *h*_*l*_(***x***) during the training process implements the function

$$h_{l}\left(x\right)=f\left(W_{l}^{T}\times x_{l}+b_{l}\right)\;1\leq l\leq L-1,$$ (3)

where ***x***_*l*_ is the input vector, ***W***_*l*_ is the weight matrix, ***b***_*l*_ is the bias vector, and *f* denotes the activation function.

Rather than take the output of the *l*th layer *h*_*l*_(***x***) as the input of the *l* + 1th layer ***x***_*l*+1_, we pass *h*_*l*_(***x***) through Eq 4 to perform dropout.

$$x_{l+1}=dropout\left(h_{l},p_{drop}\right),$$ (4)

where *p*_*drop*_ is the probability of dropout and is generally taken as 50%. After being set to zero with the probability *p*_*drop*_, *h*_*l*_(***x***) can be taken as the input of the *l* + 1th layer.

In the last layer of the network, the softmax function generates the distribution of the model *D*_*L*_(***x***) as follows:

$$D_{L}\left(x\right)=softmax\left(W_{L}^{T}\times x_{L}+b_{L}\right).$$ (5)

In the test phase, instead of setting half of the outputs to zero randomly, the network contains all the hidden units but halves the outgoing weights to offset the losses of the neurons set to zero during the training phase. This network is called the “mean network” and it can learn the general features of a model. The “mean network” is a reasonable approximation to averaging the predictions of all the dropped-out models which sample half the neurons to act on the output. When the geometric mean of all the dropped-out models and the average of the “mean network” are similar, the dropout network performs better.

With dropout, the model can sample half the neurons to act on the output. After many iterations, the units have the same probability of being extracted. Thus, the situation in which two units work together in every iteration in the training phase will not exist, and cooperation between units can be avoided. The effect of the stochastic neurons makes the classification less reliant on arbitrary units, which ameliorates overfitting.

## Proposed mixout units

We propose adopting a modified maxout unit, namely, the mixout unit, and we demonstrate that the mixout unit improves the pooling ability of the maxout unit.

### Motivation

The better use of the model-averaging ability of dropout by maxout units can be attributed partly to the pooling ability. While a maxout unit takes the maximal feature in the feature subspace that consists of *k* affine feature mappings, the output is invariant when the feature subspace changes with the random connections caused by dropout. The pooling operation on the feature subspace improves the invariant properties of the maxout units. Thus, improving the pooling ability of the maxout units can improve the feature's expressivity and descriptive ability.

**Fig 2:**
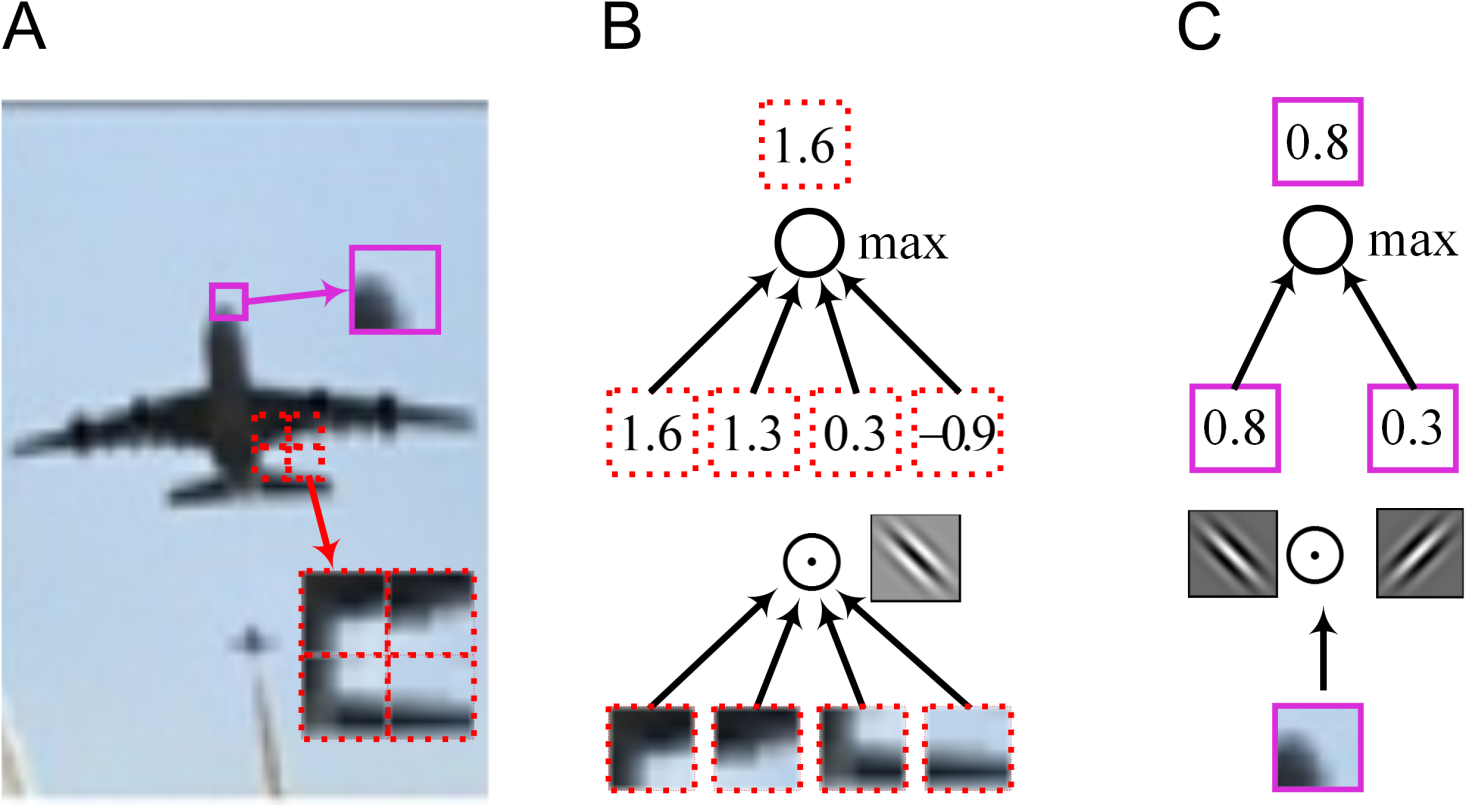
Processing flows of spatial max pooling and the maxout unit.

The processing flow of the maxout unit is shown in Fig 2C. The sub-block in the purple box in Fig 2A is taken as the input data. Two convolutional linear transformations are applied to the input, obtaining 2 different features, 0.8 and 0.3. The two features of the same input comprise the feature subspace. The maxout unit chooses the larger value (0.8) of the subspace as the output. Thus, the output describes only feature 1; it ignores feature 2.

Spatial max pooling is an operation that occurs during the subsampling stages of the CNN. The flow of spatial max pooling is given in Fig 2B. The 4 sub-blocks in the red dotted boxes in Fig 2A are taken as the inputs. One convolutional linear transformation is applied to each of the four inputs to obtain 4 different values, 1.6, 1.3, 0.3 and -0.9. The maximum (1.6) of these four will be taken as the output by the max pooling operation.

The maxout unit operates on the “feature subspace,” which comprises several affine feature mappings that are extracted by applying several convolutional linear transformations to the same input. Spatial max pooling pools over “spatial space,” which consists of the same feature mappings that are gained by applying one convolutional linear transformation on several different inputs.

The sufficient use of the model-averaging technique of dropout by maxout units partly depends on the pooling ability. Thus, improving the subspace pooling ability is helpful when combining with the model-averaging technique of dropout.

**Fig 3:**
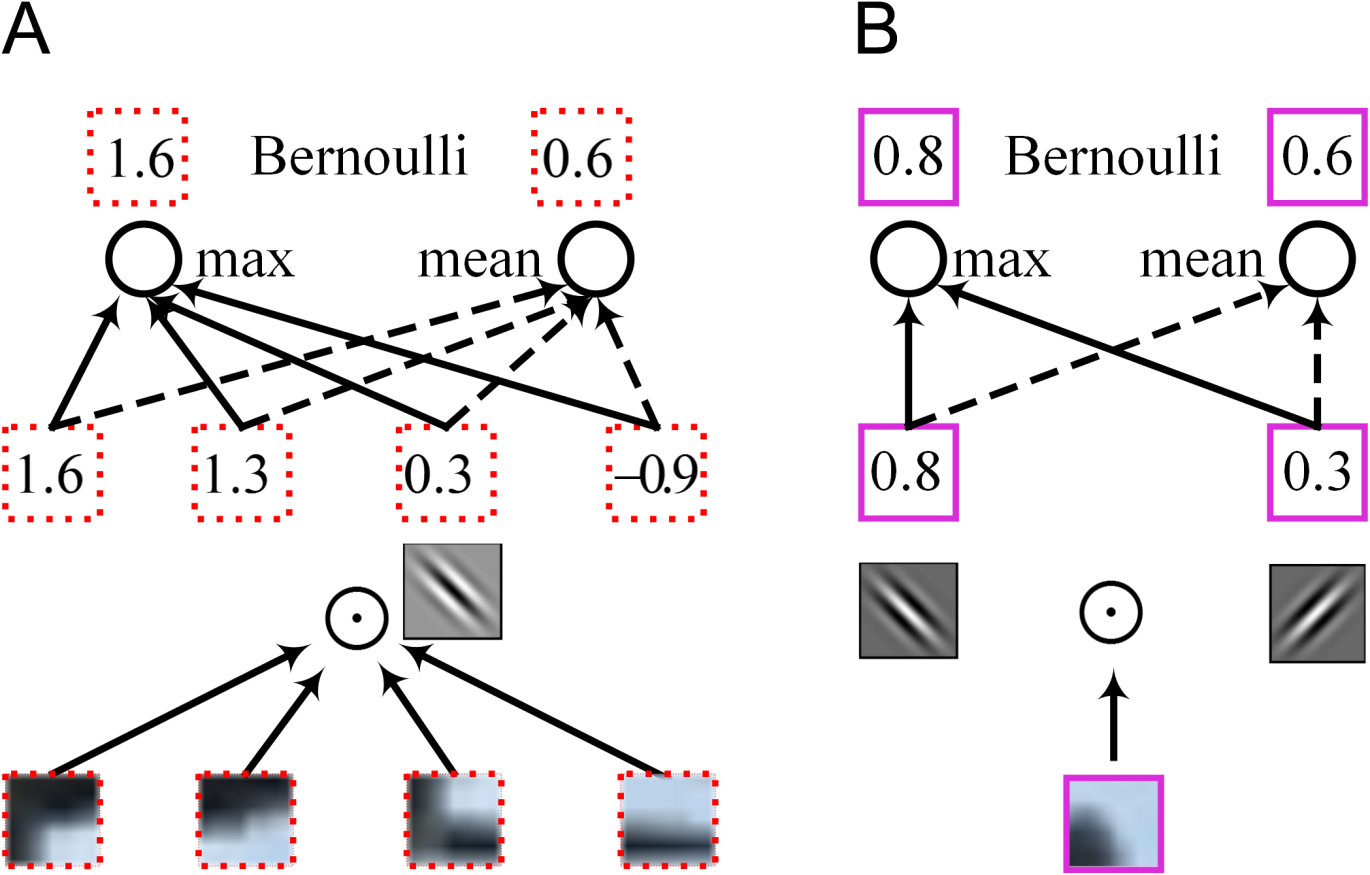
Processing flows of spatial max pooling and the mixout unit.

Spatial mixed pooling is another pooling method used in the subsampling stage [12]. In [12], the authors replaced the deterministic pooling operations with a stochastic procedure that weights the conventional max pooling and average pooling values by a Bernoulli distribution. The processing flow of spatial mixed pooling in the subsampling stage is given in Fig 3A. The 4 sub-blocks in the red dotted boxes in Fig 2A are taken as the inputs. One convolutional linear transformation is applied to the inputs, thus obtaining 4 different values, 1.6, 1.3, 0.3 and -0.9, in 4 different areas that correspond to the same convolutional kernel. Then, the maximum (1.6) and the expected value (0.6) of the four are sampled by a Bernoulli distribution. The stochastic nature of the mixed pooling method outputs the non-maximum randomly, thus improving the pooling performance.

According to the desirable properties of spatial mixed pooling, the performance of the maxout unit can be improved if we combine it with the spatial mixed pooling operation. Thus, we propose the mixout unit as an adaptive activation function.

### Mixout unit scheme

Given an input vector ***x*** ∈ R^*d*^ for the *l*th layer in a deep network, the linear feature mapping ***z*** is defined as Eq (2). By applying *k* convolutional linear transformations to the same input ***x***, *k* features are extracted, which comprise a feature subspace. We calculate the exponential probability *p*_*i*_ for the *i*th linear feature in this subspace as follows:

$$p_{i}=\frac{e^{z_{i}}}{\sum_{1}^{k}e^{z_{j}}},$$ (6)

where *z*_*i*_ is the value of the *i*th convolutional linear transformation. Then, according to the exponential probabilities, we compute $\tilde{E}$, the expected value of the feature subspace, as

$$\tilde{E}=\sum_{1}^{k}p_{i}\times z_{i}.$$ (7)

Obviously, the expected value according to the exponential probabilities can express the generality of the feature mappings subspace. More specifically, we introduce the Bernoulli distribution to achieve a balance between the maximum value and the expected value of the feature subspace. The output *h*(*x*) can be defined as follows:

$$h\left(x\right)=\lambda \underset{i\in [1,k]}{\max}z_{i}+\left(1-\lambda \right)\tilde{E},$$ (8)

where *λ* obeys the Bernoulli distribution,

$$P\left\{\lambda =k\right\}=p^{k}{\left(1-p\right)}^{1-k},k=0,1.$$ (9)

The parameter *λ* is a random value that is either 0 or 1 and indicates whether the maximum value or the expected value of the feature subspace is selected.

### Pooling ability of mixout units

The processing flow of mixout units is illustrated in Fig 3B. The sub-block in the purple box in Fig 2A is taken as the input data *x*. Two convolutional linear transformations are applied to the input *x* according to Eq 2 to obtain the two convolutional linear transformational values *z*_1_ = 0.8 and *z*_2_ = 0.3, which comprise the feature subspace. We compute the exponential probabilities of each feature mapping with Eq 6, obtaining 0.6 and 0.4. The expected value of the subspace is computed by Eq 7, yielding 0.6. We weight the maximum and expected values as shown in Eq 8 to obtain the output.

Obviously, the maximum of the feature subspace describes the maximal feature, and the expected value describes the general feature of the sub-block. According to the randomness of the Bernoulli distribution, after many iterations, the outputs of the mixout units will describe not only the maximal feature but also other details, thus improving the pooling ability.

## Experiments and analysis

In this section, we assessed the performance of the mixout units from two aspects. The first aspect addresses an analysis of the pooling ability of the mixout units. We designed a simple CNN model and analyzed the pooling ability of mixout units, maxout units [9] and probout units [1] both qualitatively and quantitatively. The second aspect addresses the performance analysis of the general mixout model. We designed the mixout-units-based NiN model (M-NiN) based on the frequently used NiN model [13] and compared its performance with models based on maxout units, probouts units, ReLU and its variants. We used an Intel (R) Core (TM) i5-4590 processor and an AMD Radeon HD 7000 series graphics card for our experiments.

### Analysis of the pooling ability of mixout units

**Fig 4:**
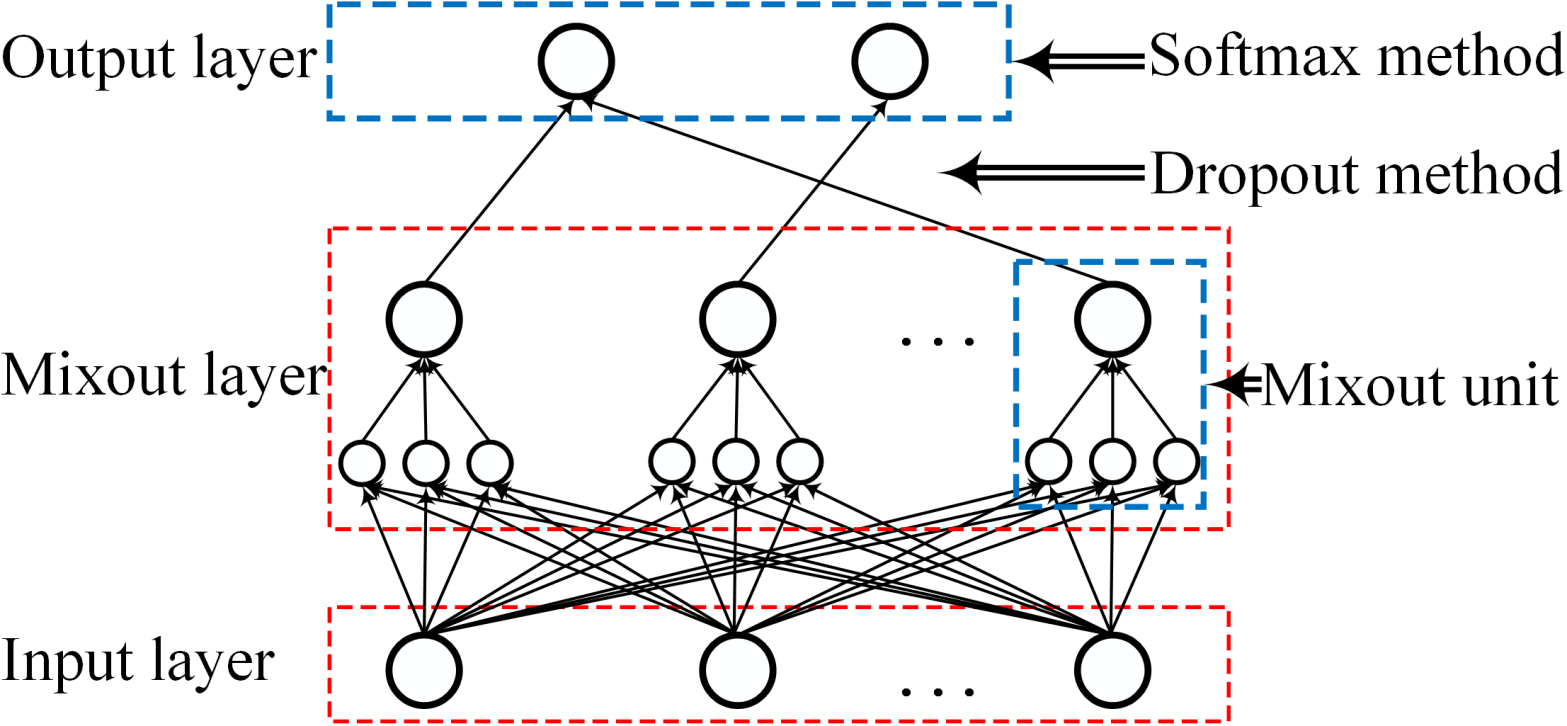
Structure of the sample network.

Because our main task is to explore the pooling ability of mixout units, we designed a simple network with only one mixout layer to avoid the potential influence of other factors such as model depth. As shown in Fig 4, this simple network consists of one input layer, one mixout layer and one output layer. The number of mixout units is 100. The connection between the final layer and the penultimate layer is processed by dropout with a probability of 50%. We then replaced the mixout units with maxout units and probout units while keeping other parameters the same for comparison and analysis.

**Fig 5:**
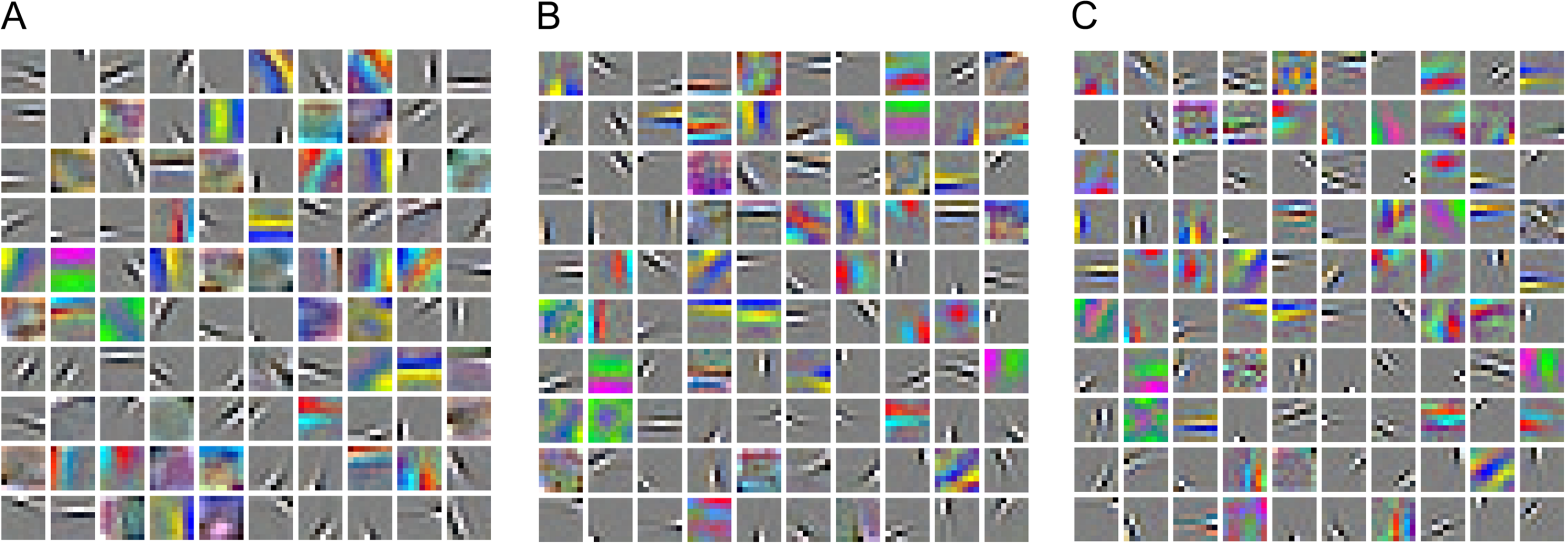
Features learned by the three networks.

We evaluated the model on the CIFAR-10 [14] dataset (http://www.cs.utoronto.ca/~kriz/cifar.html), which consists of 60,000 color images drawn from 10 classes that can be split into 50,000 training images and 10,000 test images. Objects such as animals, cars and so on appear in the center of the images. Each class has 5,000 training images and 1,000 testing images. The resolution of the images is 32×32. We visualized the first layer filters learned by the maxout, probout and mixout models, which are shown in Fig 5A, 5B and 5C, respectively. As Fig 5 shows, compared with the maxout and probout models, features learned by the mixout model are general and learns the better edge and detail features.

**Fig 6:**
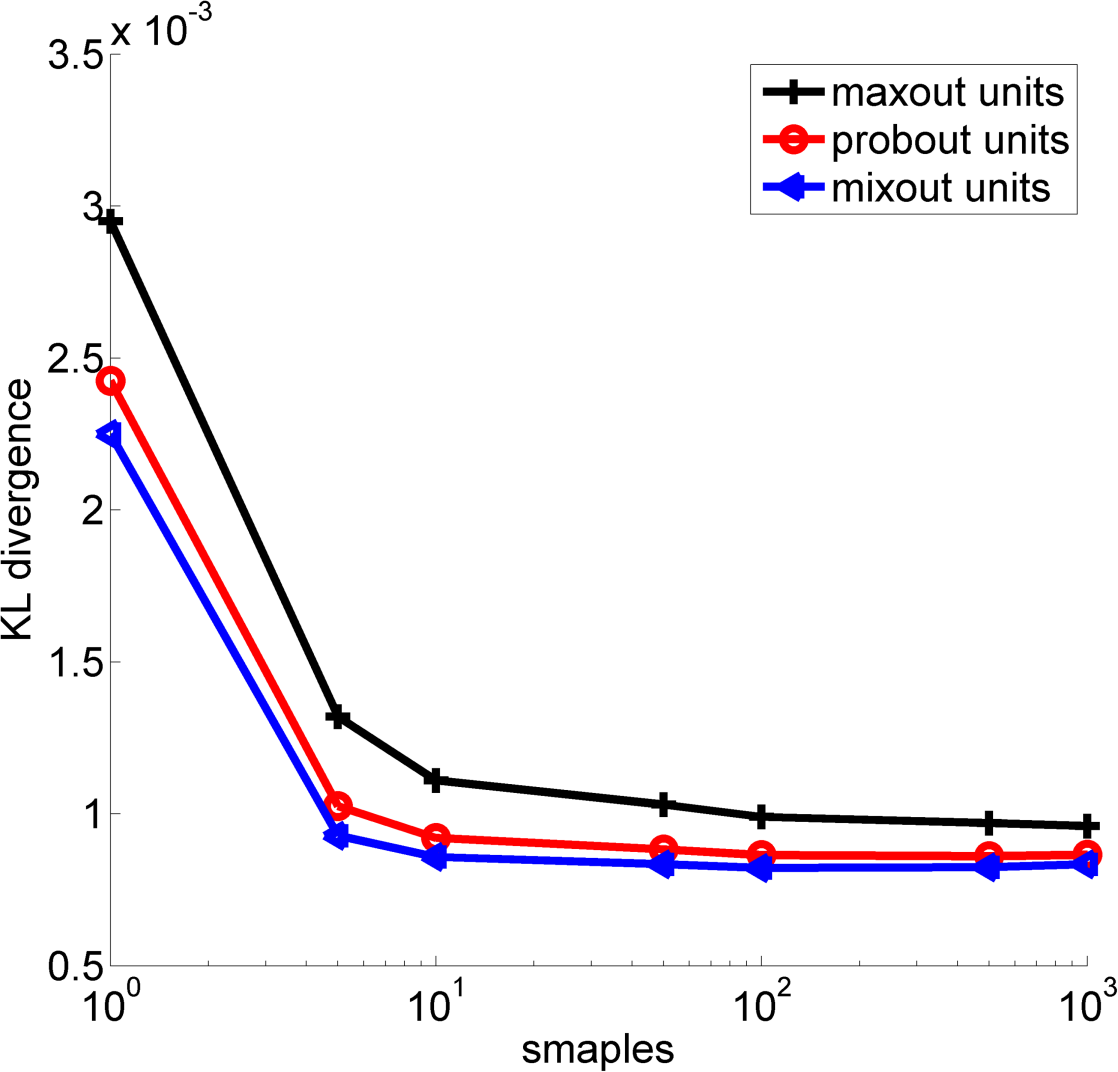
The KL divergences of the three networks.

The Kullback-Leibler (KL) divergence is a good tool for describing the differences between two distributions. It is defined as

$$KL=\sum_{x\in \Omega }p\left(x\right)\log\frac{p\left(x\right)}{q\left(x\right)},$$ (10)

where *x* is the input data that belongs to domain Ω, and *p* and *q* are two distributions about *x*. Usually, *p* is the real distribution, and *q* is the approximate distribution. The larger the value of *KL* is, the greater the difference is between the approximate distribution *q* and the real distribution *p*. Conversely, the smaller the value of *KL* is, the more similar the two distributions are. We used the average distribution of the “mean network” as the real distribution *p*, and the geometric mean of the distributions predicted by several sampled models with dropout as the approximate distribution *q*. The *KL* values of the maxout, probout and mixout models are depicted in Fig 6. The origin figure file is shown as **S1 Matlab**.

From Fig 6, we can see that the *KL* divergence between the three models decreases as the number of samples increases. This finding suggests that dropout does indeed perform model averaging. Moreover, the approximation is more accurate for the mixout units than for the maxout and probout units, which indicates that the mixout units make better use of the model-averaging technique of dropout. This finding suggests that the mixout units improve the pooling ability of the maxout units.

### Performance analysis of general mixout models

To further analyze the mixout units, we assessed the performance of mixout-units-based models. The performances of commonly used CNN models such as AlexNet [4], NiN [13] and ResNet [15] differ due to their structure and depth. Because the NiN model performs well and is easy to implement, we designed a mixout-units-based NiN (M-NiN) model for experiments.

#### Data sets

We evaluated our proposed M-NiN model on three different image classification datasets, CIFAR-10, CIFAR-100 [14] (http://www.cs.utoronto.ca/~kriz/cifar.html) and SVHN [16] (http://ufldl.stanford.edu/housenumbers/). CIFAR-100 is an extension of CIFAR-10; however, it can be divided into 100 classes in which each class includes 500 training images and 100 test images. The SVHN dataset is a collection of images depicting digits that were obtained from Google street view images. It consists of more than 600,000 color images at a resolution of 28×28 that can be classified according to the digits in the images, which range from 0 to 9.

By considering the overall characteristics of the three datasets, we know that each class in the CIFAR-100 dataset has the fewest images (600) and that each class in the CIFAR-10 dataset has ten times that many (6,000). Moreover, in SVHN, each class contains 60,000 images—ten times the number in the CIFAR-10 dataset. In other words, these three datasets can represent general datasets and validate the applicability of M-NiN to some extent.

#### M-NiN model

**Fig 7:**
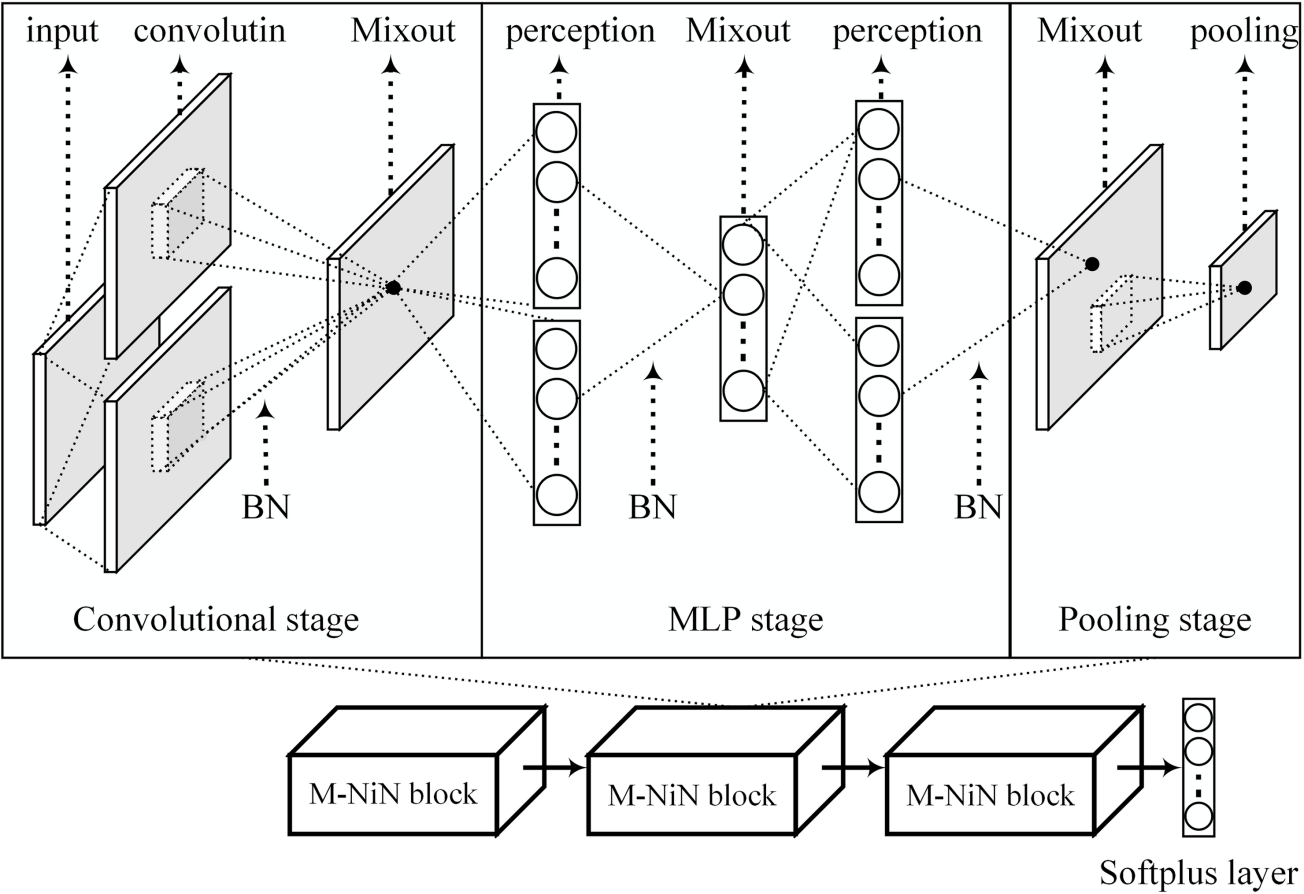
The architecture of the proposed M-NiN model.

Each block in the NiN consists of three stages: a convolutional stage, a multi-layer perceptron (MLP) stage and a pooling stage. Our M-NiN model replaced ReLU activation with mixout units for nonlinear transformation, as shown in Fig 7. The M-NiN model includes three stacked M-NiN blocks and one softmax layer. As with the general NiN block, each M-NiN block also consists of three stages: a convolutional stage, a mixout-based MLP (MMLP) stage and a pooling stage. We added batch normalization (BN) after every convolutional and perception layer.

The parameters of the M-NiN are shown in Table 1. All three convolutional layers in the three blocks have 192 mixout units, with kernels of 5×5, 5×5 and 3×3. Convolution is a linear transformation over different areas; thus, there is no subspace, and the dimension is 0. The number of padding pixels on the border is 2, and the stride pixel is 1. Every M-NiN block has 2 mixout unit-based MLP (MMLP) layers. We set the number of mixout units in the first MMLP of the three blocks to 160, 192 and 192 and in the second MMLP of the three blocks to 96, 192 and 160. Because the mixout units operate over several features of the same input, the kernel is 1×1, and the number of features in one subspace is 5. There is no padding pixel, and the stride pixel is 1. Each block has one pooling layer. The three layers in the three blocks are set as max pooling, max pooling and average pooling with kernels of 3×3, 3×3 and 8×8, respectively. The pooling operates on the same feature; thus, there is no subspace. The number of padding pixels is 2, and the striding pixels were set to 2, 2 and 1. We processed the connection between the last layer of the block and the first layer of the next block at a probability of 50%. In the softmax layer, which is used for classification, the numbers were set to 10, 100 and 10 for the three benchmark datasets, respectively. Dropout was used in the connection between the last layer of the block and the softmax layer at a probability of 50%.

**Table 1:** Parameters of the M- NiN model.

Layer	Number of Mixout units	Kernel size	Subspace dimension	Padding pixel	Striding pixel
Convolutional layer	192	5×5	0	2	1
MMLP -1	160	1×1	5	0	1
MMLP-2	96	1×1	5	0	1
Pooling layer	Max pooling	3×3	0	2	2
Dropout 0.5					
Convolutional layer	192	5×5	0	2	1
MMLP -1	192	1×1	5	0	1
MMLP -2	192	1×1	5	0	1
Pooling layer	Max pooling	3×3	0	2	2
Dropout 0.5					
Convolutional layer	192	3×3	0	2	1
MMLP -1	192	1×1	5	0	1
MMLP -2	160	1×1	5	0	1
Pooling layer	average pooling	8×8	0	2	1
Dropout 0.5					
Softmax	Classification 10/100				

The maxout-units-based NiN model and the probout-units-based NiN model are similar to M-NiN. We designed the structure of M-NiN and set the parameters according to [1] [9] [12] and[17]. The structures and parameters of ReLU-[18], LReLU[19]-, and PReLU[20]-based NiNs were set according to [12][21][22] and [23].

#### Experimental method

We performed cross-validation on the three datasets using a training method and validation-splitting measure similar to that described by Goodfellow et al. [9]. For the classification task on CIFAR-10, we pre-processed the data by global contrast normalization and ZCA whitening. We then selected the hyper-parameters of the model by minimizing the error on 40,000 images randomly selected from the training set. Next, we recorded the value of the log likelihood on the last 10,000 examples at the point of minimal validation error with the selected hyper-parameters. We retrained the model on the full training set from scratch and stopped when the new likelihood matched the old one. We followed a similar procedure on CIFAR-100 as with CIFAR-10.

For the experiment on SVHN, we chose 73,257 training images and 20,032 test images as well as 531,131 additional, but somewhat less difficult, examples. Then, we selected 400 digits per class for training and 200 digits per class for cross-validation. Finally, we retrained the model on the entire set of 598,388 digits.

We trained the maxout-units-, probout-units- and mixout-units-based models using the methods described above, and we trained the ReLU-, LReLU- and PReLU-based NiN models using the methods described in [23].

#### Experimental results and analysis

Table 2 lists the results of the experiments. Overall, the error rate on CIFAR-100 is the highest, and the error rate on SVHN is the lowest, revealing that all the models perform better when applied to a larger dataset. This result occurs because by their nature, larger datasets help prevent overfitting due to the enormous amounts of experimental data they contain. Models based on the maxout units and their variants, the probout and mixout units, achieve higher accuracy than models based on ReLU and its variants, LReLU- and PReLU. Of the three datasets, the lowest error rate among all the six models was obtained by M-NiN. This finding suggests that the mixout-based models perform better.

**Table 2:** Error rates of the different models on the three datasets.

models	CIFAR-10	CIFAR-100	SVHN
ReLU	12.45	42.90	2.78
LReLU	11.20	42.04	2.69
PReLU	11.79	41.63	2.51
Maxout units	11.68	38.57	2.47
Probout units	11.35	38.14	2.39
Mixout units	11.02	37.39	2.31

**Fig 8:**
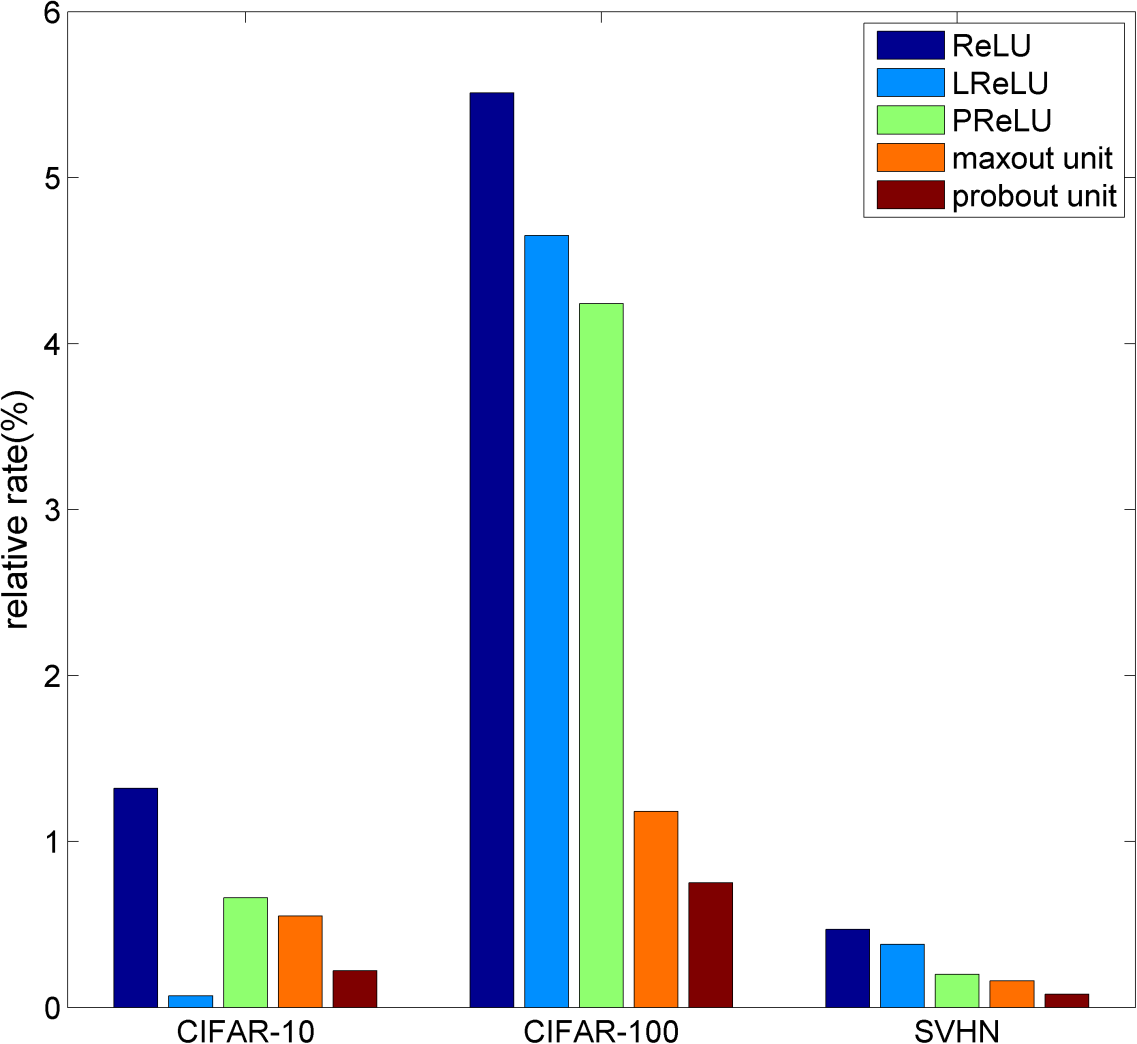
Error rates of the other models compared with M-NiN.

To further analyze the classification ability of M-NiN, we computed the relative error rates of the other five types of models on the three datasets compared with the M-NiN model; these results are shown in Fig 8. The origin figure file is shown as **S2 Matlab**.

As Fig 8 shows, the differences between M-NiN and the other five types of models are relatively larger in CIFAR- 100 as compared with CIFAR-10 and SVHN. That means that when used on a relatively small dataset, M-NiN performs much better than the other models. The reason is that mixout units can regulate the data by sufficient use of the model-averaging technique of dropout, effectively preventing overfitting, and that, in addition, larger datasets with more examples inherently ameliorate overfitting to some extent. Compared with the maxout network, M-NiN reduces the error rate by 5.5%, 11.8% and 1.6% on the three datasets. This finding suggests that the mixout model has better feature and classification abilities.

**Fig 9:**
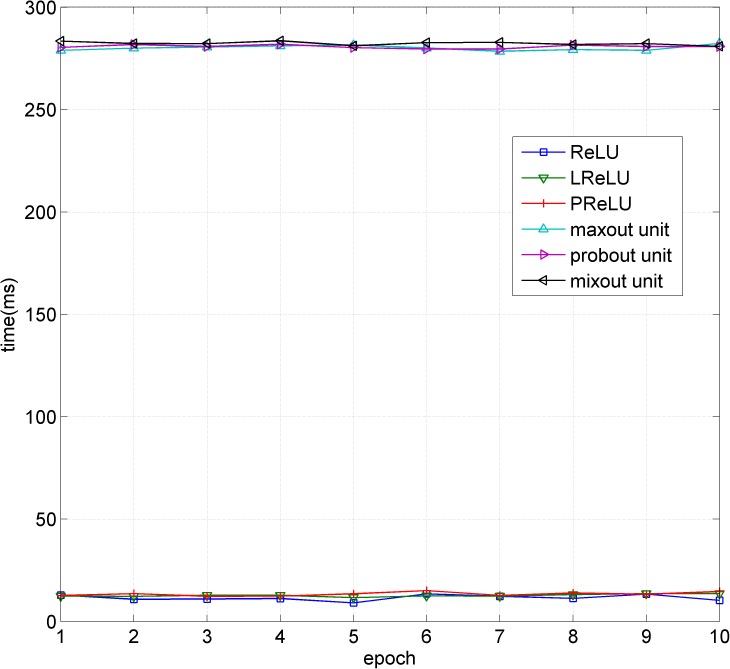
Time consumption comparison of the six models.

Compared with the maxout unit, the mixout unit adds two steps: calculating the expected value and weighting it and using the maximum with a Bernoulli distribution. The added steps increase the complexity of the model. However, considering the complexity of the model and the calculation of the parameters, the additional steps do not consume much extra time. Using CIFAR-10 as an example, we recorded the time consumption of the first ten epochs of the six models as shown in Fig 9. The origin figure file is shown as **S3 Matlab**. The experimental data of Table 2 can be taken as a reference for comparison in this paper.

From Fig 9, we can obtain that the difference in the time consumption among the M-NiN, maxout model and probout model is not significant. Overall, models based on the maxout unit and its variants consume more time than those based on ReLU and its variants because the maxout unit family is more complex than the ReLU family in the nonlinear transformation.

## Conclusions

In this paper, we presented the mixout unit as an adaptive activation function. We designed a sample model for pooling ability analysis as well as a general model for performance analysis of mixout units. The mechanism for mixing the maximum and average of the mixout units improves the subspace pooling operation, thus leading to a better utilization of the model-averaging ability of dropout. We conducted several experiments on three benchmark datasets. The results revealed the desirable properties of mixout units. Because sufficient use of the model-averaging technique of dropout prevents overfitting to some extent, mixout units have unsatisfactory performances on large datasets, which somewhat constrains the overfitting effect. Interesting avenues for future work include applying mixout units to other network architectures such as VGG or ResNet.

## Supporting information

S1 Matlab The KL divergences of the three networks. (FIG)

S2 Matlab Error rates of the other models compared with M-NiN. (FIG)

S3 Matlab Time consumption comparison of the six models. (FIG)
